# The Effects of High-Definition Transcranial Alternating Current Stimulation (HD-tACS) on Dual-Task Performance and Upper-Limb Multi-Joint Motor Control

**DOI:** 10.3390/life16091424

**Published:** 2026-08-27

**Authors:** Wei Zhuang, Zhifei Zhang, Xiuling Bian, Keyi Yin

**Affiliations:** 1 School of Physical Education, Hangzhou Normal University, Hangzhou 311121, China; zhangzhifei_2027@163.com; 2 School of Physical Education, Chengdu University, Chengdu 610106, China; bianxiuling@cdu.edu.cn; 3 Key Laboratory of Sports Technology Analysis and Skill Assessment, Xi’an Physical Education University, General Administration of Sport, Xi’an 710068, China; 4 School of Sport and Health Science, Xi’an Physical Education University, Xi’an 710068, China

**Keywords:** high-definition transcranial alternating current stimulation (HD-tACS), cognitive–motor dual task, dual-task performance, upper-limb motor control, N-back task

## Abstract

This study investigated the effects of high-definition transcranial alternating current stimulation (HD-tACS) on cognitive–motor dual-task performance and upper-limb multi-joint motor control. Sixteen right-handed healthy male participants completed a double-blind, randomized crossover experiment involving active tACS and sham tACS conditions separated by a one-week washout interval. A 4 × 1 HD-tACS montage was applied over the left dorsolateral prefrontal cortex at 5 Hz for 20 min. Participants performed a synchronous cognitive–motor dual-task paradigm that combined an N-back working memory task with an upper-limb throwing task under different task difficulty levels. Task performance outcomes included accuracy, reaction time, release velocity, peak velocity, movement duration, and dual-task cost. Upper-limb motor control outcomes included elbow and wrist joint range of motion, peak angular velocity, and peak joint flexion and extension moments. Accuracy showed a significant Stimulation × Time interaction. Under active stimulation, adjusted accuracy increased by 5.4 percentage points in N1 and 10.1 percentage points in N2, whereas the corresponding changes under sham stimulation were not significant. Reaction time, release velocity, peak velocity, and movement duration showed no significant stimulation-related changes. Release-velocity DTC also showed a significant Stimulation × Time interaction across N1 and N2. Under active stimulation, the signed DTC shifted upward, indicating a smaller release-velocity decrement relative to N0. The task-specific follow-up was significant for N2 but not N1; however, the Stimulation × Time × Task interaction was not significant. Most upper-limb kinematic and kinetic outcomes showed no significant stimulation-related changes. Active HD-tACS was associated with higher accuracy and a favorable shift in release-velocity DTC, while most motor, kinematic, and kinetic outcomes were unchanged. The effects were limited to selected behavioral outcomes and did not differ significantly between N1 and N2.

## 1. Introduction

Transcranial electrical stimulation (tES) has been investigated as a non-invasive approach for modulating neural activity related to cognition and motor control [1]. Among tES protocols, transcranial alternating current stimulation (tACS) applies weak alternating currents at a specified frequency and intensity and may interact with endogenous neural oscillations within targeted frequency bands [1]. Experimental evidence suggests that tACS can influence neuronal excitability [2,3] and may induce after-effects related to neural plasticity, depending on the stimulation parameters [4,5]. Accordingly, tACS has been applied across different frequency bands to modulate cognitive processes [6,7]. In the motor domain, beta-frequency tACS over the motor cortex has been reported to affect finger movements [8,9]. These findings suggest that tACS may influence both cognitive and motor functions. However, most evidence concerning motor performance has been obtained from relatively simple movements. Whether these effects extend to multi-joint actions remains unclear.

Many daily activities require cognitive processing and motor execution to operate together. These demands are commonly examined using cognitive–motor dual-task (DT) paradigms [10]. Under DT conditions, the cognitive and motor components may compete for limited processing resources [10], resulting in slower responses [11], poorer balance control [12,13], or greater gait variability [14,15]. Herold et al. [10] classified dual tasks as sequential or simultaneous according to whether the cognitive and motor components are performed concurrently. Simultaneous DT paradigms can be further described as additive or integrated. In additive paradigms, the cognitive task imposes an additional load but is not required to complete the motor task. In integrated paradigms, cognitive information determines the appropriate motor response and is therefore necessary for successful task completion.

Integrated DT paradigms may better represent daily activities in which perception, decision-making, and movement execution contribute to the same behavioral goal [10]. Performance in such tasks depends on the processing of relevant information, selection of the correct response, and execution of the required movement [11,16]. A stimulation-related change may therefore be expressed in response accuracy or reaction time, motor performance, or both [9,13]. Studies of visually guided reaching and fine-visuomotor performance also indicate that executive demands and speed–accuracy trade-offs may be expressed differently across accuracy, response-time, and movement outcomes [17,18]. These findings support the separate assessment of task performance, dual-task cost, and movement execution rather than treating integrated DT performance as a single outcome.

However, most studies of tACS and cognitive–motor dual-task performance have involved older adults or used additive dual-task designs [13]. It remains unclear whether tACS can affect performance in an integrated dual task. In this type of task, cognitive processing directly guides the motor response. It is also unclear whether any effect is limited to task accuracy or is accompanied by changes in motor performance and movement control.

Therefore, this study aimed to investigate the effects of active HD-tACS, compared with sham stimulation, on performance during an integrated cognitive–motor dual-task involving an N-back task and a multi-joint throwing action. Task performance, dual-task cost, and upper-limb kinematic and kinetic outcomes were assessed.

## 2. Methods

### 2.1. Participants

The minimum required sample size was estimated using G*Power software version 3.1.9.4. No single primary outcome was prespecified because the study was designed to examine stimulation-related changes across several domains, including cognitive-task performance, motor-task performance, dual-task cost, and upper-limb kinematic and kinetic outcomes. Accordingly, the power analysis was not based specifically on N-back accuracy or any other individual outcome. Instead, it was conducted to provide a sample-size estimate for the planned repeated-measures design. Assuming an alpha level of 0.05, a statistical power of 0.80, and a large effect size of Cohen’s f = 0.40, corresponding to a partial η^2^ of approximately 0.14, the minimum required sample size was estimated to be 10 participants [19].

Seventeen male participants were initially recruited. Participants were undergraduate and graduate students recruited from a sports university and had sports-training backgrounds. One participant was excluded because of a family history of epilepsy; therefore, 16 male participants were included in the final experiment. All participants were right-handed and had no previous experience with transcranial electrical or magnetic stimulation. All participants confirmed that they had not previously participated in studies involving N-back tasks. Before formal testing, they received standardized instructions regarding the N-back procedure and were provided with sufficient practice time to become familiar with the task and fully understand the experimental requirements. Their demographic characteristics are presented in Table 1. All participants were informed of the experimental procedures and provided written informed consent before participation.

Before the formal experiment, all participants completed a medical history screening. The exclusion criteria were a personal or family history of psychiatric disorders, epilepsy, or headache; use of neurological or psychiatric medication within the previous six months; and shoulder injury or incomplete recovery from shoulder injury within the previous six months. Participants were instructed to avoid alcohol, caffeine, and strenuous physical activity and to maintain adequate sleep for 72 h before each experimental session. All procedures were conducted in accordance with current implementation guidelines for transcranial electrical stimulation [20].

### 2.2. Experimental Design

This study used a double-blind, randomized crossover design. The order of active and sham stimulation was generated using a computer-based random allocation program. Before the experiment, the two stimulation modes were coded as Stimulation 1 and Stimulation 2. The condition code was accessible only to the investigator operating the electrical stimulation device. Participants and all investigators responsible for task administration, outcome assessment, and data processing remained unaware of the stimulation identity throughout testing and data analysis. Each participant visited the laboratory twice, with a one-week washout interval between visits to reduce potential carryover effects [21,22]. All post-stimulation assessments were completed within 30 min after stimulation to reduce the possibility that the stimulation effect would diminish over time [4,23].

**Figure 1:**
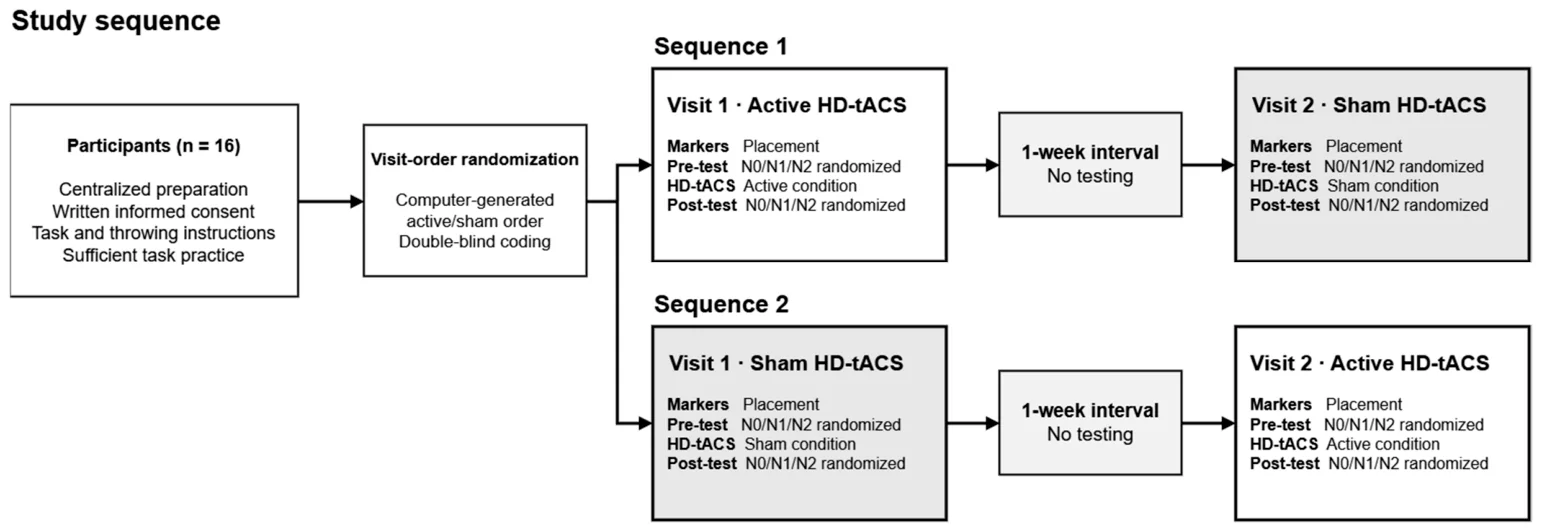
Overall experimental schedule of the randomized, double-blind crossover protocol.

Before formal testing, all participants provided written informed consent and completed a centralized preparatory session that included standardized instructions regarding the experimental procedures, task requirements, and throwing technique, followed by sufficient task practice. The formal experiment employed a randomized, double-blind crossover design comprising two visits separated by a 1-week interval. Each participant received active HD-tACS during one visit and sham HD-tACS during the other, with the visit sequence determined using a computer-generated randomization schedule. At each visit, motion-capture markers were placed before participants completed the pre-stimulation assessment. The N0, N1, and N2 conditions were administered in a randomized order. Participants then received active or sham HD-tACS targeting the left dorsolateral prefrontal cortex, followed immediately by the post-stimulation assessment. During the post-stimulation assessment, the N0, N1, and N2 conditions were again administered in a randomized order using the same task procedures. All post-stimulation assessments were completed within 30 min after stimulation. An overview of the experimental design and testing sequence is presented in Figure 1.

### 2.3. Experimental Setup

**Figure 2:**
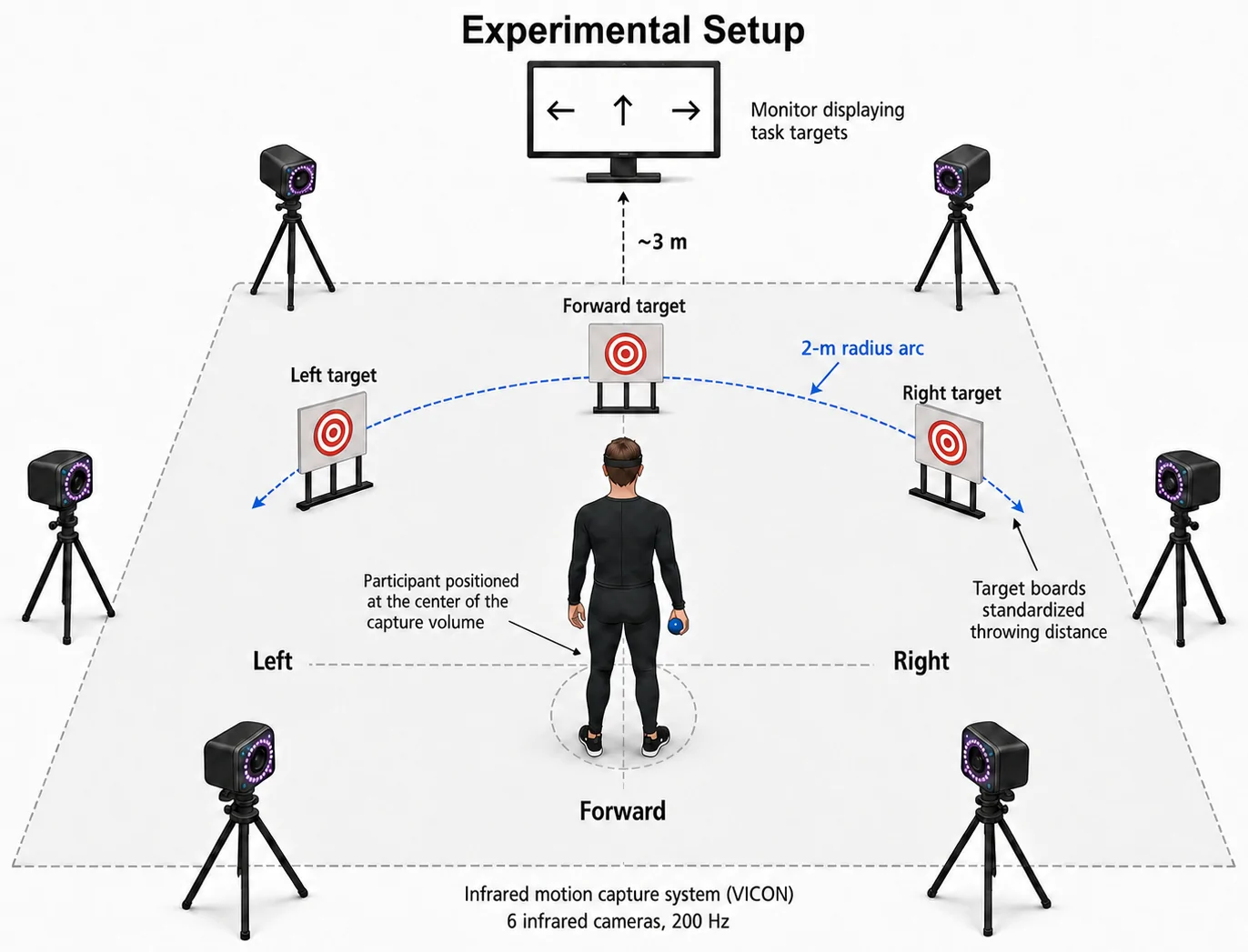
Experimental setup for data collection.

Biomechanical data were recorded using a six-camera Vicon infrared motion-capture system (Vicon Motion Systems Ltd., Oxford, UK) at a sampling frequency of 200 Hz. Participants stood at a standardized location at the center of the calibrated capture volume, with their initial stance and body orientation kept consistent across all trials. Three target boards were positioned in front of, to the left of, and to the right of the participant at an equal radial distance of 2 m from the starting position. This arrangement ensured a consistent throwing distance across the three target directions. A monitor positioned approximately 3 m in front of the participant displayed the task cues.The experimental setup for biomechanical data collection is shown in Figure 2.

**Figure 3:**
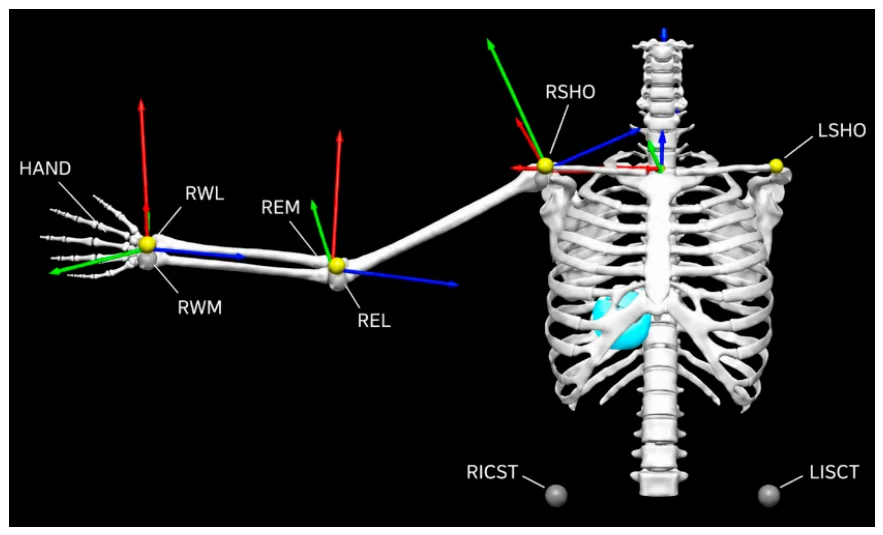
Anatomical marker placement for motion-capture analysis.

To construct a segmental kinematic model of the trunk and right upper limb, nine spherical reflective markers, each 14 mm in diameter, were attached to anatomical landmarks according to standard model construction procedures. Marker placement was performed by the same investigator across testing sessions to minimize placement variability. A static calibration trial was recorded before the experimental trials to establish the segmental model. The global coordinate system was defined with the X-axis aligned with the forward throwing direction. The anatomical marker placement for motion-capture analysis is illustrated in Figure 3. The anatomical landmarks and corresponding marker abbreviations are presented in Table 2.

### 2.4. Synchronous Dual-Task Paradigm

A synchronous cognitive–motor dual-task paradigm was developed by adding a throwing action to the conventional N-back framework. Participants were required to process and retain directional information according to the applicable N-back rule and then use this information to select the target direction for each throw. Thus, cognitive processing and motor-response selection were integrated within the same trial.

The paradigm comprised three conditions with increasing working-memory demands: N0, N1, and N2. N0 served as the low-load reference condition. In the N0 condition, participants threw the object toward the direction indicated by the currently displayed arrow. In the N1 condition, they threw toward the direction indicated by the arrow presented one trial earlier. In the N2 condition, they threw toward the direction indicated by the arrow presented two trials earlier.

**Figure 4:**
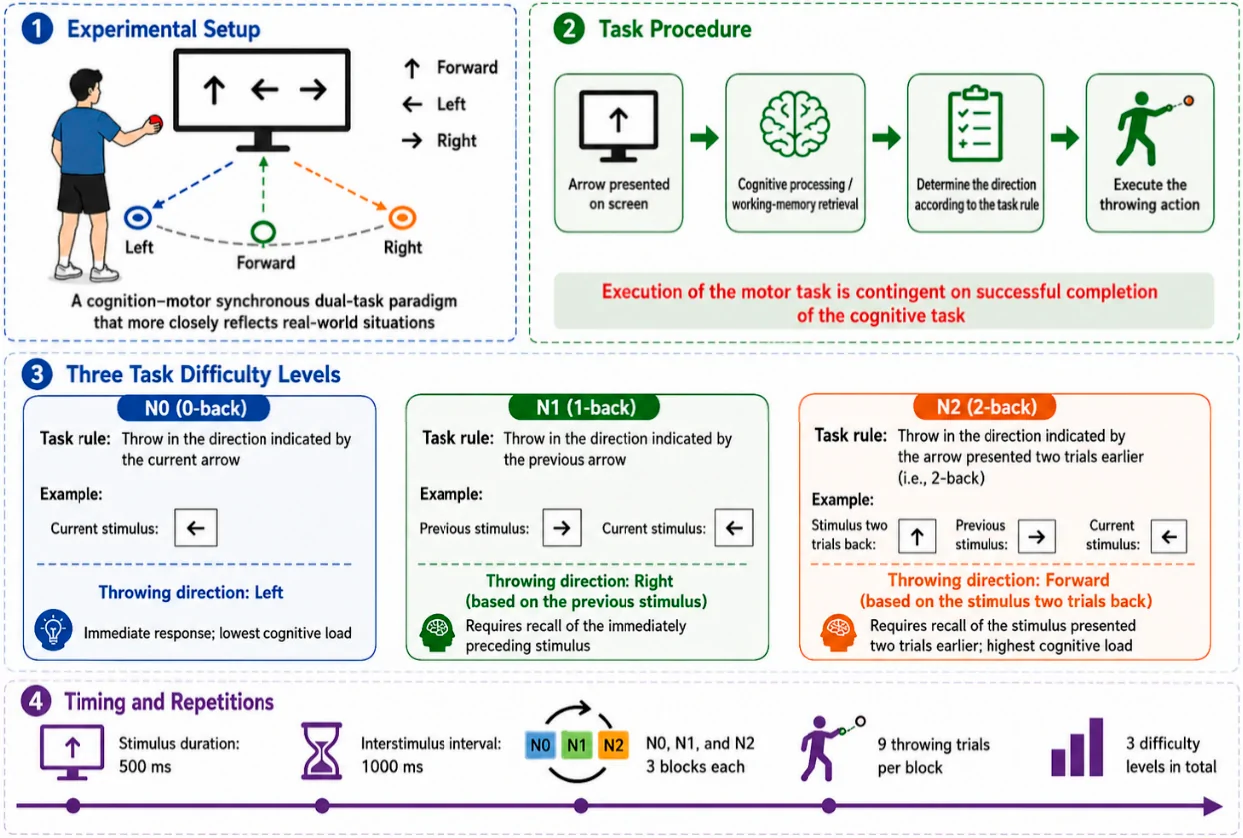
Schematic workflow of the synchronous cognitive–motor dual-task paradigm.

**Figure 5:**
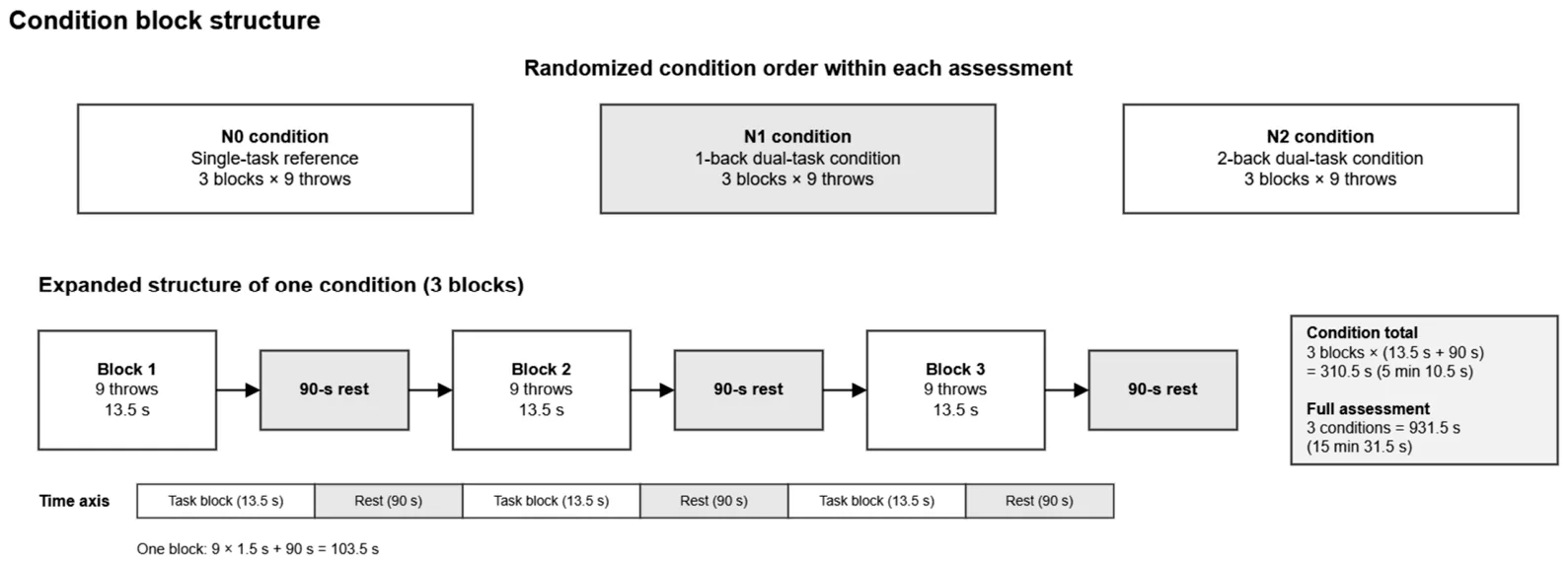
Structure of one pre- or post-stimulation assessment.

Directional arrows pointing forward, left, or right were presented on a monitor in a pseudorandomized sequence. The sequences were arranged so that the three required throwing directions were equally represented within each condition. Each arrow was displayed for 500 ms, followed by a 1000 ms interstimulus interval, resulting in a total trial duration of 1500 ms. Following stimulus onset, participants applied the relevant N-back rule, selected the required target direction, and executed the corresponding throw as quickly and accurately as possible. Each condition comprised three blocks of nine scored throwing trials, resulting in 27 trials per condition and 81 trials per pre- or post-stimulation assessment. Across the three blocks of each condition, each throwing direction was required nine times. A 90 s rest interval was provided after each block to reduce cognitive and physical fatigue. The temporal relationship among cue presentation, memory processing, and throwing execution is illustrated in Figure 4. The detailed procedure and components of the pre- or post-stimulation assessment are outlined in Figure 5.

To standardize the throwing movement, participants were instructed to perform each throw using a continuous and controlled movement while minimizing excessive shoulder extension, irregular wrist flicking, and substantial trunk rotation. These instructions were intended to reduce compensatory movements and improve the consistency of the recorded kinematic data. Participants were required to prioritize both response speed and directional accuracy.

Trial validity was determined separately from cognitive-response accuracy. A throw directed toward a target that was inconsistent with the applicable N-back rule was classified as an incorrect cognitive response. Such trials were retained in the denominator for accuracy calculations and were not repeated. In contrast, a trial was considered procedurally invalid and was repeated when data acquisition was interrupted, motion-capture data were incomplete, the participant failed to execute a continuous throwing movement because of clear movement interruption, the landing distance was grossly outside the acceptable range, or another identifiable procedural or technical error occurred. The invalid attempt was excluded and replaced by the repeated trial.

### 2.5. tACS Protocol

**Figure 6:**
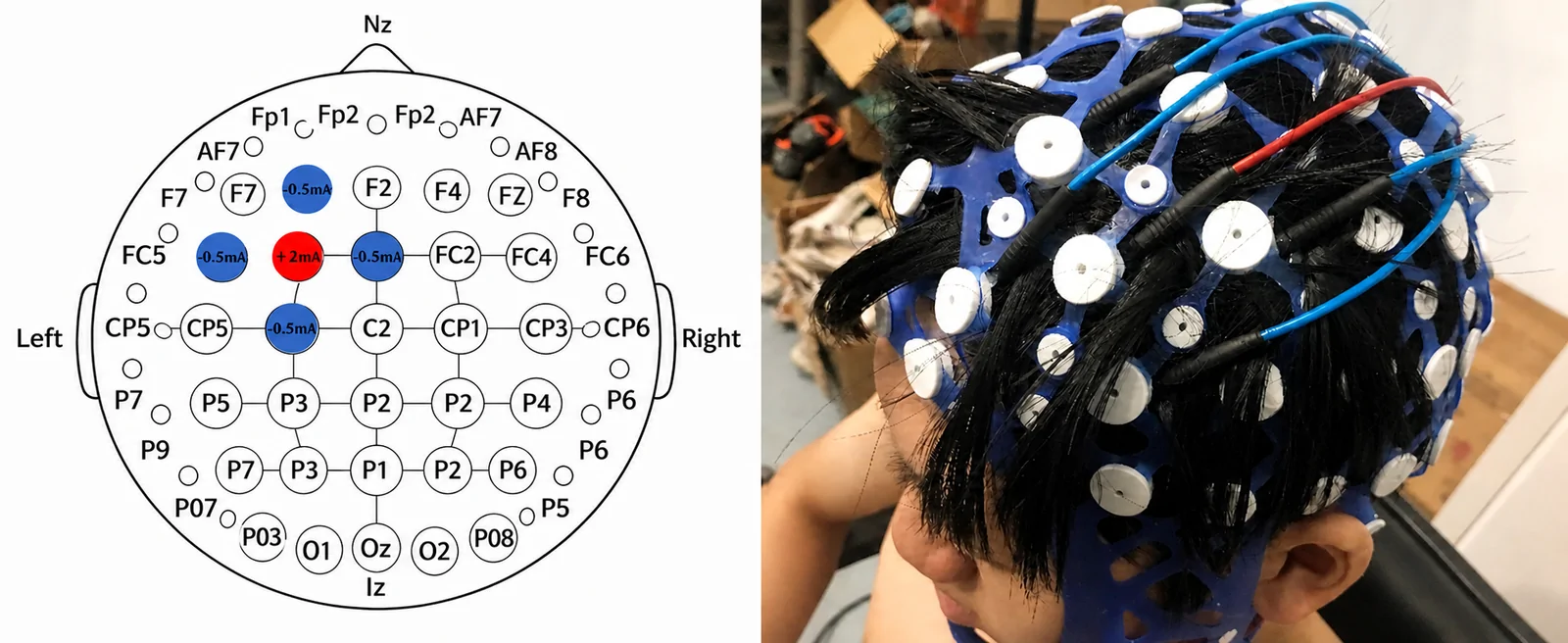
Electrode montage for the stimulation protocol.

Transcranial alternating current stimulation was delivered using a neuroConn transcranial electrical stimulator (neuroConn GmbH, Ilmenau, Germany). During stimulation, participants remained seated quietly and did not perform any cognitive or motor tasks. To target the left dorsolateral prefrontal cortex, a 4 × 1 electrode montage was arranged according to the international 10–20 electroencephalography system. The central electrode was positioned over F3, and the four surrounding return electrodes were positioned over FC3, F5, AF3, and F1. Figure 6 shows the electrode montage used for the stimulation protocol.

In the active condition, 5 Hz alternating current was delivered with a peak amplitude of 2 mA at the central electrode, corresponding to 4 mA peak-to-peak. The total return current was distributed equally across the four surrounding electrodes, with a nominal peak amplitude of 0.5 mA at each electrode. Active stimulation consisted of a 30 s ramp-up period, 20 min of stimulation at the target intensity, and a 30 s ramp-down period. Sham stimulation was administered using the same electrode montage and procedural duration, with the device’s built-in sham mode selected by the stimulation operator. The 5 Hz frequency and F3-centered montage were selected based on the involvement of prefrontal theta activity in working-memory processing and previous studies applying theta-frequency tACS over the prefrontal cortex [24,25].

Five circular rubber electrodes, each 1.6 cm in diameter, were applied using conductive EEG paste. Electrode impedance was continuously monitored throughout stimulation and maintained below 10 kΩ. Participants were monitored during and after stimulation, and any participant-reported discomfort or adverse effects were recorded.

### 2.6. Data Processing and Outcome Measures

The anterior–posterior, mediolateral, and vertical directions were defined as the X-, Y-, and Z-axes, respectively. Local coordinate systems were established for the upper-limb segments from the anatomical standing posture, and a standard skeletal model was constructed in Visual3D (C-Motion, Germantown, MD, USA). Three-dimensional motion-capture data were imported into Visual3D and smoothed using a second-order Butterworth low-pass filter with a cutoff frequency of 7 Hz. Visual3D Pipeline scripts were then used to identify the relevant movement events and calculate the outcome variables.

The outcome measures were categorized into task-performance variables and upper-limb motor-control variables. Dual-task performance variables were calculated using trials from all three throwing directions. Because throwing mechanics may differ across directions, only correctly performed forward throws with complete motion-capture data were included in the kinematic and kinetic analyses.

Because N0 required no working-memory updating, it served as the single-task reference for calculating dual-task costs under N1 and N2. N0 outcome values were not entered as an additional task level in the primary inferential analyses, which focused on the N1 and N2 conditions. Where applicable, dual-task costs were calculated relative to N0. The definitions, units, and abbreviations of all outcome measures are provided in Table 3.

### 2.7. Statistical Analysis

All analyses were performed using IBM SPSS Statistics, version 25.0 (IBM Corp., Armonk, NY, USA). The N1 and N2 conditions were included jointly in the models as two levels of the factor Task. The N0 condition was excluded from the primary stimulation-effect models and served strictly as a matched reference to calculate dual-task costs (DTC).

Accuracy (number of correct responses out of 27 trials) was analyzed using a binomial generalized linear mixed-effects model with a logit link. All continuous outcomes—including reaction time, velocity metrics, movement duration, DTC measures, and upper-limb kinematics and kinetics—were analyzed using linear mixed-effects models fitted by restricted maximum likelihood. Missing observations were excluded on an analysis-by-analysis basis without imputation.

For all models, Participant was included as a random intercept. Fixed effects included Sequence, Period, TaskPosition, Stimulation, Time, and Task, along with their relevant interactions (Stimulation × Time, Stimulation × Task, Time × Task, and Stimulation × Time × Task). Sequence and Period were included to account for crossover sequence and visit-related effects, respectively. We used Type III tests of fixed effects and Kenward–Roger denominator degrees of freedom.

As no single primary outcome was prespecified, all analyses were considered exploratory. The primary test for the stimulation effect was the Stimulation × Time interaction, while the Stimulation × Time × Task interaction tested whether the stimulation-related changes differed between N1 and N2.

To control for multiple comparisons, the Benjamini–Hochberg false discovery rate (FDR) procedure was applied separately to the Stimulation × Time tests across three prespecified outcome families: (1) task performance, (2) DTC outcomes, and (3) upper-limb motor control. Interactions surviving FDR correction were followed by Holm-adjusted simple-effects comparisons. Results are reported using F statistics, denominator degrees of freedom, unadjusted *p* values, FDR-adjusted *q* values, adjusted estimated marginal means (or probabilities), and 95% confidence intervals, with statistical significance set at 0.05.

## 3. Results

### 3.1. Dual-Task Performance

#### 3.1.1. Accuracy

**Figure 7:**
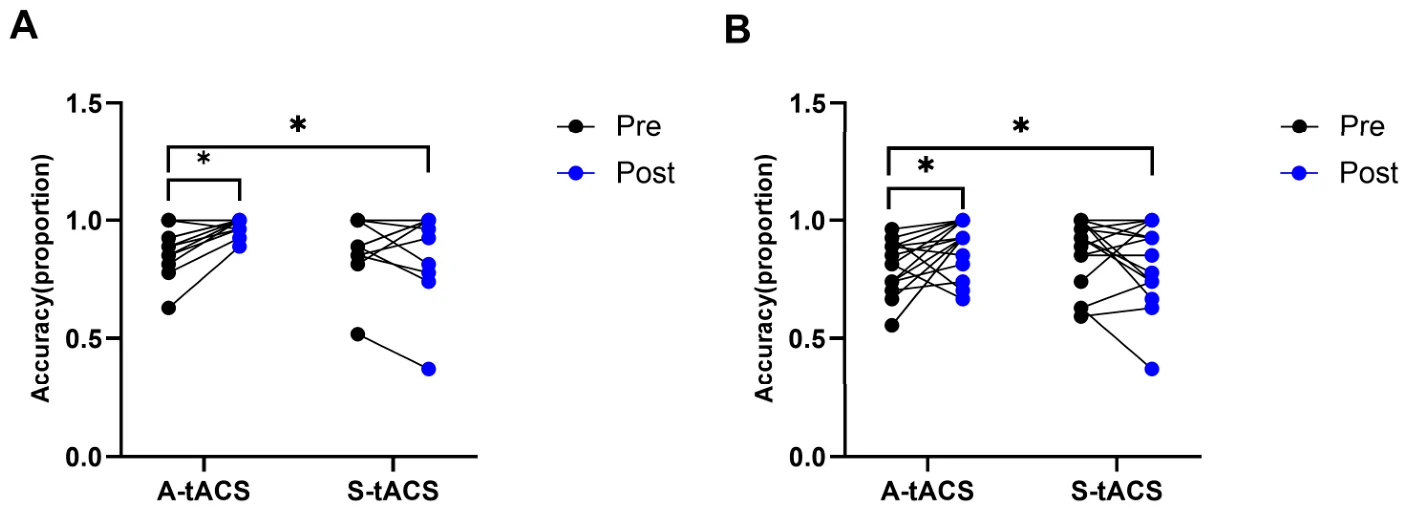
Changes in accuracy before and after active and sham HD-tACS in the N1 and N2 tasks: (**A**) N1; (**B**) N2. Note. The study included 16 participants. Lines connect the pre- and post-stimulation values obtained from the same participant. Asterisks indicate statistically significant Holm-adjusted pre-to-post comparisons (* adjusted *p* < 0.05). The overall analysis did not show that the stimulation-related change differed significantly between the N1 and N2 tasks.

Accuracy demonstrated a significant Stimulation × Time interaction, F(1, 116) = 29.50, *p* < 0.001, *q* < 0.001. The corresponding Stimulation × Time×task interaction, however, was not significant, F(1, 116) = 1.07, *p* = 0.303. Under active stimulation, adjusted accuracy increased from 0.934 to 0.988 in N1 (Δ = 0.054, 95% CI [0.013, 0.094], Holm-adjusted *p* = 0.012; Table 4) and from 0.808 to 0.909 in N2 (Δ = 0.101, 95% CI [0.049, 0.153], Holm-adjusted *p* < 0.001; Table 4). Conversely, no significant changes were observed under sham stimulation (N1: *p* = 0.671; N2: *p* = 0.121). Because the three-way interaction was nonsignificant, these task-specific estimates should not be interpreted as evidence of differing stimulation effects between N1 and N2.The adjusted probabilities and 95% CIs are presented in Table 5. Additionally, a significant period effect was observed, F(1, 116) = 28.42, *p* < 0.001 (Table 6). The changes in accuracy before and after active and sham HD-tACS in the N1 and N2 tasks are presented in Figure 7.

**Table 4:** Follow-up comparisons for accuracy based on adjusted probabilities.

Task	Stimulation	Pre EMM [95% CI]	Post EMM [95% CI]	Post − Pre	95% CI for Change	Holm-Adjusted *p*	Interpretation
N1	Sham	0.944 [0.886, 0.974]	0.938 [0.874, 0.970]	−0.006	[−0.036, 0.023]	0.671	Not significant
N1	Active	0.934 [0.868, 0.968]	0.988 [0.968, 0.995]	0.054	[0.013, 0.094]	0.012 *	Significant increase
N2	Sham	0.871 [0.812, 0.914]	0.831 [0.760, 0.884]	−0.040	[−0.092, 0.011]	0.121	Not significant
N2	Active	0.808 [0.733, 0.866]	0.909 [0.862, 0.941]	0.101	[0.049, 0.153]	<0.001 *	Significant increase

*N* = 16. Values are adjusted probabilities [95% CI]. * Holm-adjusted *p* < 0.05 for within-condition pre-to-post comparisons. Note: The overall analysis showed no significant difference in stimulation-related changes between N1 and N2; therefore, task-specific comparisons do not imply differing stimulation effects between the two tasks.

**Table 5:** Adjusted estimated marginal means (95% confidence intervals) for task-performance outcomes.

Outcome	Task	Sham Pre	Sham Post	Active Pre	Active Post
Accuracy (proportion)	N1	0.944 [0.886, 0.974]	0.938 [0.874, 0.970]	0.934 [0.868, 0.968]	0.988 [0.968, 0.995]
Accuracy (proportion)	N2	0.871 [0.812, 0.914]	0.831 [0.760, 0.884]	0.808 [0.733, 0.866]	0.909 [0.862, 0.941]
Reaction time (s)	N1	0.866 [0.791, 0.948]	0.818 [0.748, 0.895]	0.793 [0.725, 0.859]	0.767 [0.701, 0.839]
Reaction time (s)	N2	0.788 [0.721, 0.863]	0.760 [0.694, 0.832]	0.812 [0.742, 0.889]	0.746 [0.681, 0.816]
Release velocity (m/s)	N1	1.458 [1.042, 1.874]	1.364 [0.948, 1.780]	1.384 [0.974, 1.794]	1.323 [0.910, 1.737]
Release velocity (m/s)	N2	1.380 [0.965, 1.796]	1.447 [1.031, 1.863]	1.288 [0.878, 1.699]	1.348 [0.934, 1.761]
Peak velocity (m/s)	N1	2.583 [2.251, 2.914]	2.617 [2.286, 2.949]	2.587 [2.257, 2.917]	2.538 [2.207, 2.869]
Peak velocity (m/s)	N2	2.545 [2.213, 2.877]	2.632 [2.301, 2.964]	2.580 [2.249, 2.910]	2.601 [2.270, 2.932]
Movement duration (s)	N1	0.339 [0.300, 0.377]	0.340 [0.302, 0.379]	0.317 [0.278, 0.355]	0.324 [0.285, 0.362]
Movement duration (s)	N2	0.342 [0.303, 0.380]	0.339 [0.301, 0.378]	0.335 [0.296, 0.373]	0.333 [0.294, 0.371]

Note. *N* = 16. Values are adjusted marginal estimates [95% CI]. Accuracy is shown as an adjusted probability; reaction time as a back-transformed geometric mean (following log-transformation); and release velocity, peak velocity, and movement duration as adjusted marginal means. See Section 2.7 and Table 6 for statistical significance evaluation. Individual estimates should only be interpreted as significant if supported by their corresponding statistical comparisons.

**Table 6:** Statistical tests of stimulation effects on task-performance outcomes.

Outcome	Stimulation × Time F (df)	*p*	BH-FDR *q*	Stimulation × Time × Task F(df)	*p*	Other Significant Design Term
Accuracy	29.50 (1, 116)	<0.001	<0.001 *	1.07 (1, 116)	0.303	Period: F(1, 116) = 28.42, *p* < 0.001 †
Reaction time	0.04 (1, 102.53)	0.842	0.934	0.30 (1, 102.53)	0.588	None
Release velocity	0.01 (1, 95.89)	0.934	0.934	0.02 (1, 95.82)	0.894	None
Peak velocity	1.02 (1, 95.99)	0.316	0.790	0.01 (1, 95.97)	0.908	None
Movement duration	0.03 (1, 102.05)	0.865	0.934	0.02 (1, 102.05)	0.900	Period: F(1, 102.05) = 8.31, *p* = 0.005 †

Note. *N* = 16. Unadjusted *p* values and Benjamini–Hochberg FDR-adjusted *q* values are reported for the five prespecified Stimulation × Time tests. Bold text denotes statistically significant results. * *q* < 0.05 (significant after BH-FDR correction). Significant interactions were followed by Holm-adjusted comparisons. † Significant design-related effect; this should not be interpreted as evidence of a stimulation-induced pre-to-post change.

#### 3.1.2. Reaction Time

No significant stimulation × time interaction was found for reaction time, F (1, 102.53) = 0.04, *p* = 0.842, *q* = 0.934. The stimulation × time × task interaction was also nonsignificant (*p* = 0.588). Adjusted estimated marginal means and 95% confidence intervals are reported in Table 5.

#### 3.1.3. Motor Task Performance

No significant stimulation × time interactions were found for release velocity (*p* = 0.934, *q* = 0.934), peak velocity (*p* = 0.316, *q* = 0.790), or movement duration (*p* = 0.865, *q* = 0.934). Their stimulation × time × task interactions were also nonsignificant (all *p* ≥ 0.894). Adjusted means and 95% CIs for these outcomes are presented in Table 5.

Movement duration showed a significant period effect, F (1, 102.05) = 8.31, *p* = 0.005 (β = −0.056 s, 95% CI [−0.094, −0.017]; Table 6). The period effects observed for accuracy and movement duration represent visit-related rather than stimulation effects. No other sequence, period, or task-position effects were significant.

#### 3.1.4. Dual-Task Cost

**Figure 8:**
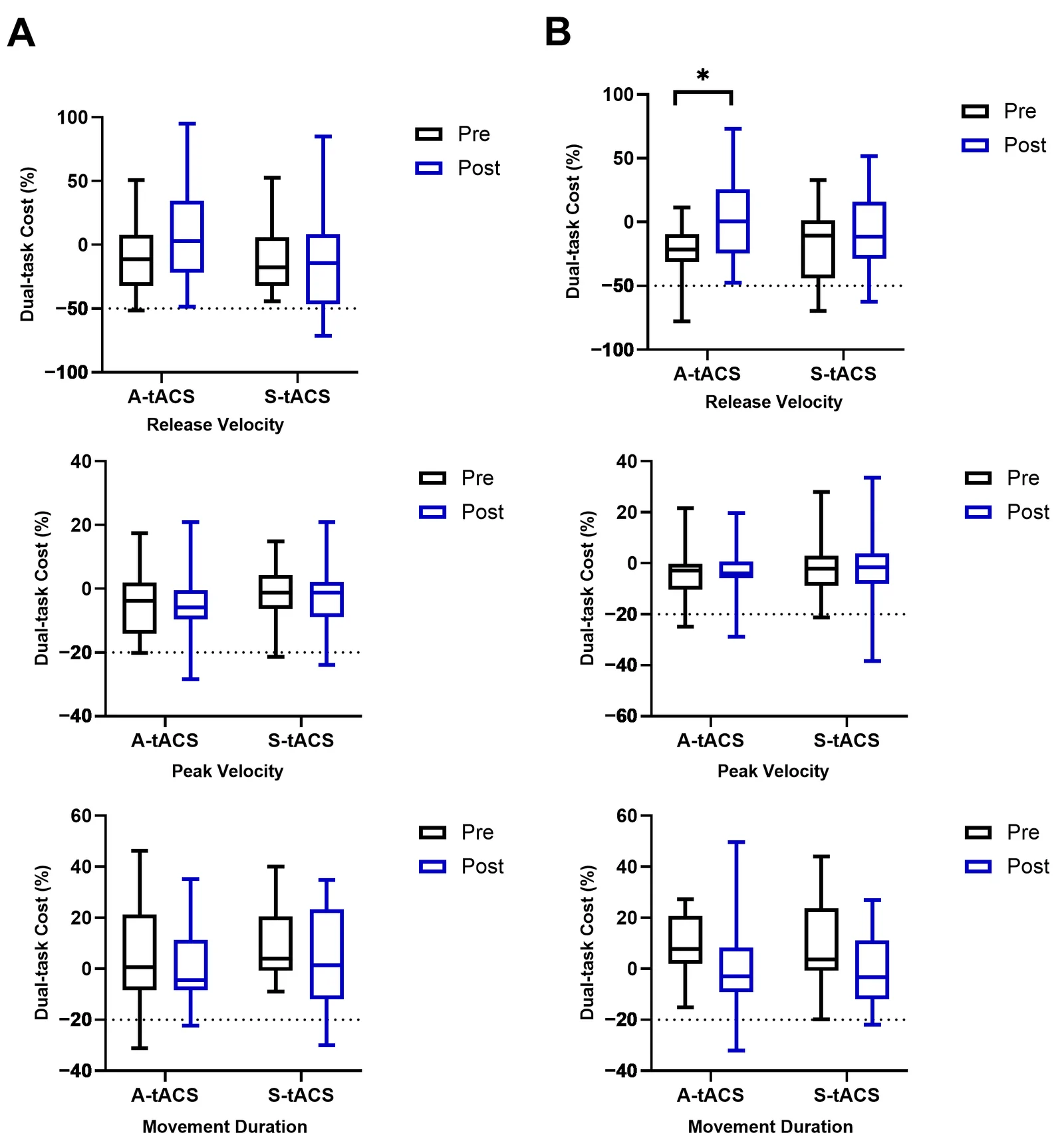
Changes in dual-task cost before and after active and sham HD-tACS in the N1 and N2 tasks: (**A**) N1; (**B**) N2. Note. *N* = 16. Boxplots summarize participant-level value distributions. * *p* < 0.05 (Holm-adjusted pre-to-post comparisons). The overall analysis showed no significant difference in stimulation-related changes between N1 and N2.

Of the three dual-task-cost (DTC) outcomes, only release-velocity DTC showed a significant Stimulation × Time interaction, F(1, 95.49) = 6.83, *p* = 0.010, which remained significant after BH-FDR correction (*q* = 0.031; Table 7). The corresponding Stimulation × Time×task interaction was not significant, F(1, 94.51) = 0.08, *p* = 0.783, indicating no evidence that the stimulation effect differed between N1 and N2. Specifically, active stimulation increased the mean release-velocity DTC across N1 and N2 by 33.06 percentage points. This effectively reducedthe velocity deficit compared to the N0 reference (Δ = 33.06 percentage points, 95% CI [15.81, 50.32], Holm-adjusted *p* < 0.001; Table 8). In contrast, no significant change occurred under sham stimulation (Δ = 1.01 percentage points, 95% CI [−16.16, 18.17], Holm-adjusted *p* = 0.908; Table 8). The changes in dual-task costbefore and after active and sham HD-tACS in the N1 and N2 tasks are presented in Figure 8.

**Table 7:** Mixed-model tests of stimulation effects on dual-task-cost outcomes.

Outcome	Stimulation × Time, F (df1, df2)	*p*	BH-FDR *q*	Stimulation × Time × Task, F (df1, df2)	*p*	Other Significant Fixed Effect
Release-velocity DTC	6.83 (1, 95.49)	*p *= 0.010	*q *= 0.031 *	0.08 (1, 94.51)	*p* = 0.783	—
Peak-velocity DTC	1.47 (1, 95.14)	*p* = 0.228	*q* = 0.287	0.02 (1, 94.26)	*p* = 0.901	Stimulation: F(1, 98.71) = 5.00, *p* = 0.028 †
Movement-duration DTC	1.15 (1, 102.14)	*p* = 0.287	*q* = 0.287	0.006 (1, 102.14)	*p* = 0.938	—

Note. *N* = 16. Unadjusted *p* values and Benjamini–Hochberg FDR-adjusted *q* values are reported for the three prespecified Stimulation × Time tests. Bold text denotes statistically significant results. * *q* < 0.05 (significant after BH-FDR correction). Significant interactions were followed by Holm-adjusted comparisons. † Another statistically significant effect that does not represent a stimulation-induced pre-to-post change.

**Table 8:** Follow-up comparisons for release-velocity dual-task cost.

Contrast Level	Stimulation	Task	Adjusted Pre [95% CI]	Adjusted Post [95% CI]	Δ Post − Pre [95% CI]	Unadjusted *p*	Holm-Adjusted *p*
Across task levels	Sham	N1 and N2	−11.46 [−25.87, 2.95]	−10.45 [−24.87, 3.96]	1.01 [−16.16, 18.17]	*p* = 0.908	*p* = 0.908
Across task levels	Active	N1 and N2	−18.46 [−32.56, −4.36]	14.60 [0.51, 28.70]	33.06 [15.81, 50.32]	*p *< 0.001	*p *< 0.001 *
Task-specific	Sham	N1	−8.99 [−27.79, 9.82]	−13.14 [−31.91, 5.63]	−4.15 [−28.52, 20.21]	*p* = 0.736	*p* = 1.000
Task-specific	Sham	N2	−13.93 [−32.70, 4.84]	−7.77 [−26.58, 11.04]	6.16 [−18.20, 30.53]	*p* = 0.617	*p* = 1.000
Task-specific	Active	N1	−12.15 [−30.66, 6.37]	12.39 [−6.15, 30.92]	24.53 [0.17, 48.89]	*p* = 0.048	*p* = 0.145
Task-specific	Active	N2	−24.78 [−43.29, −6.26]	16.82 [−1.73, 35.38]	41.60 [17.24, 65.95]	*p *= 0.001	*p *= 0.004 *

Note. *N* = 16. Values are adjusted marginal estimates [95% CI]. Averaged and task-specific comparisons were Holm-adjusted as separate families. * *p* < 0.05 (Holm-adjusted). The overall analysis showed no significant difference in stimulation-related changes between N1 and N2; thus, task-specific comparisons are purely descriptive and do not imply a task-specific stimulation effect.

In task-specific follow-up comparisons, the change remained significant for N2 (Δ = 41.60 percentage points, 95% CI [17.24, 65.95], Holm-adjusted *p* = 0.004), but not for N1 (Holm-adjusted *p* = 0.145). However, given the nonsignificant three-way interaction, these task-specific differences should not be interpreted as evidence of divergent stimulation effects between N1 and N2. Finally, no significant Stimulation × Time interactions were found for peak-velocity DTC (*p* = 0.228, *q* = 0.287) or movement-duration DTC (*p* = 0.287, *q* = 0.287), and their corresponding three-way interactions were also nonsignificant (both *p* ≥ 0.901). Although a stimulation main effect was observed for peak-velocity DTC (*p* = 0.028), this represented an average condition difference across time rather than a stimulation-induced pre-to-post change. Adjusted marginal means and 95% CIs are presented in Table 9.

### 3.2. Kinematic and Kinetic Outcomes

#### 3.2.1. Joint Range of Motion

**Figure 9:**
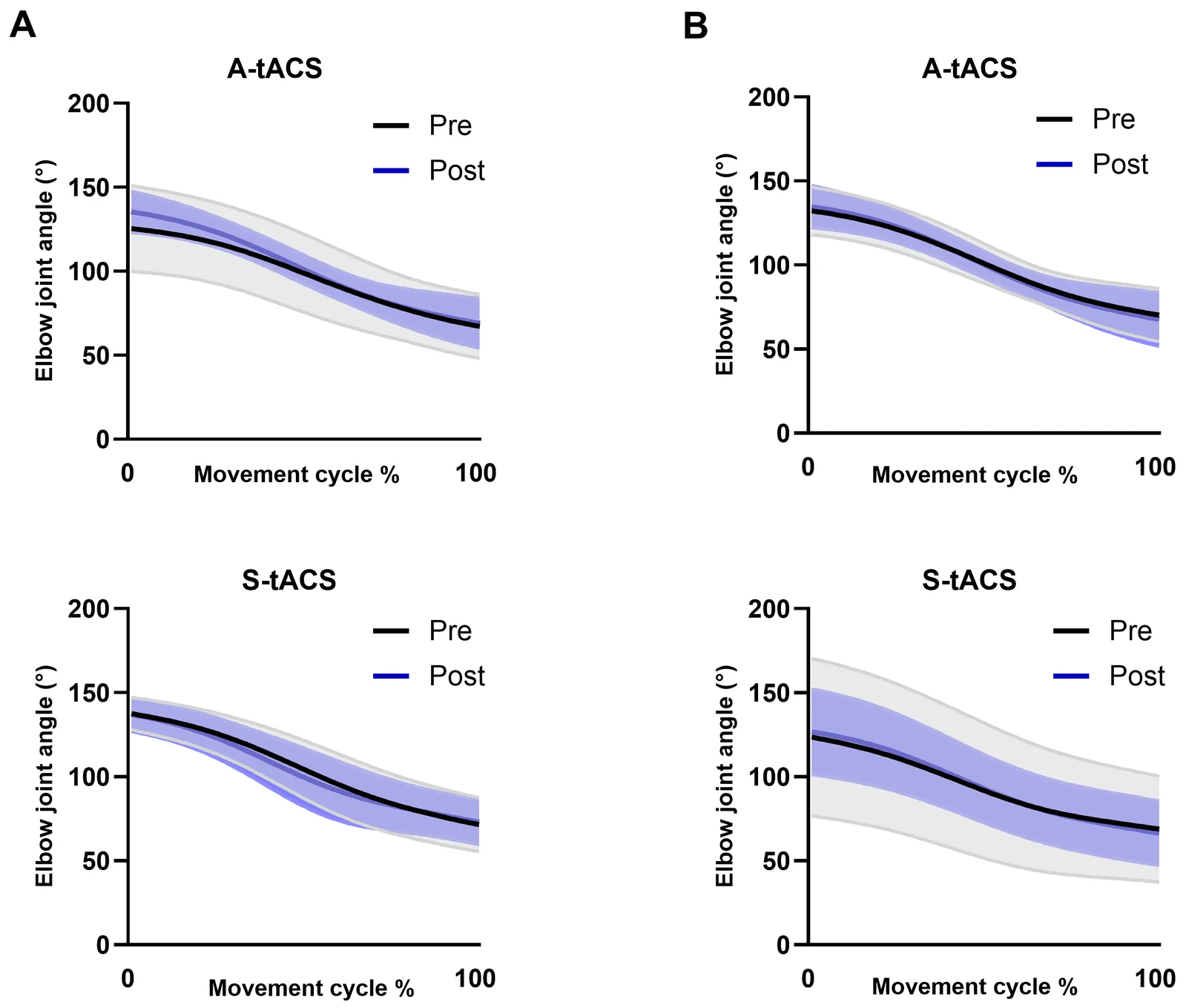
Elbow joint-angle trajectories before and after active and sham HD-tACS in the N1 and N2 tasks: (**A**) N1; (**B**) N2. Note. The study included 16 participants. Continuous curves represent group means, and the shaded regions represent standard deviations. No point-by-point statistical tests were performed on the continuous curves. Inferential analyses were based on the prespecified discrete kinematic or kinetic features extracted from the trajectories. Panels (**A**,**B**) represent the N1 and N2 tasks, respectively.

**Figure 10:**
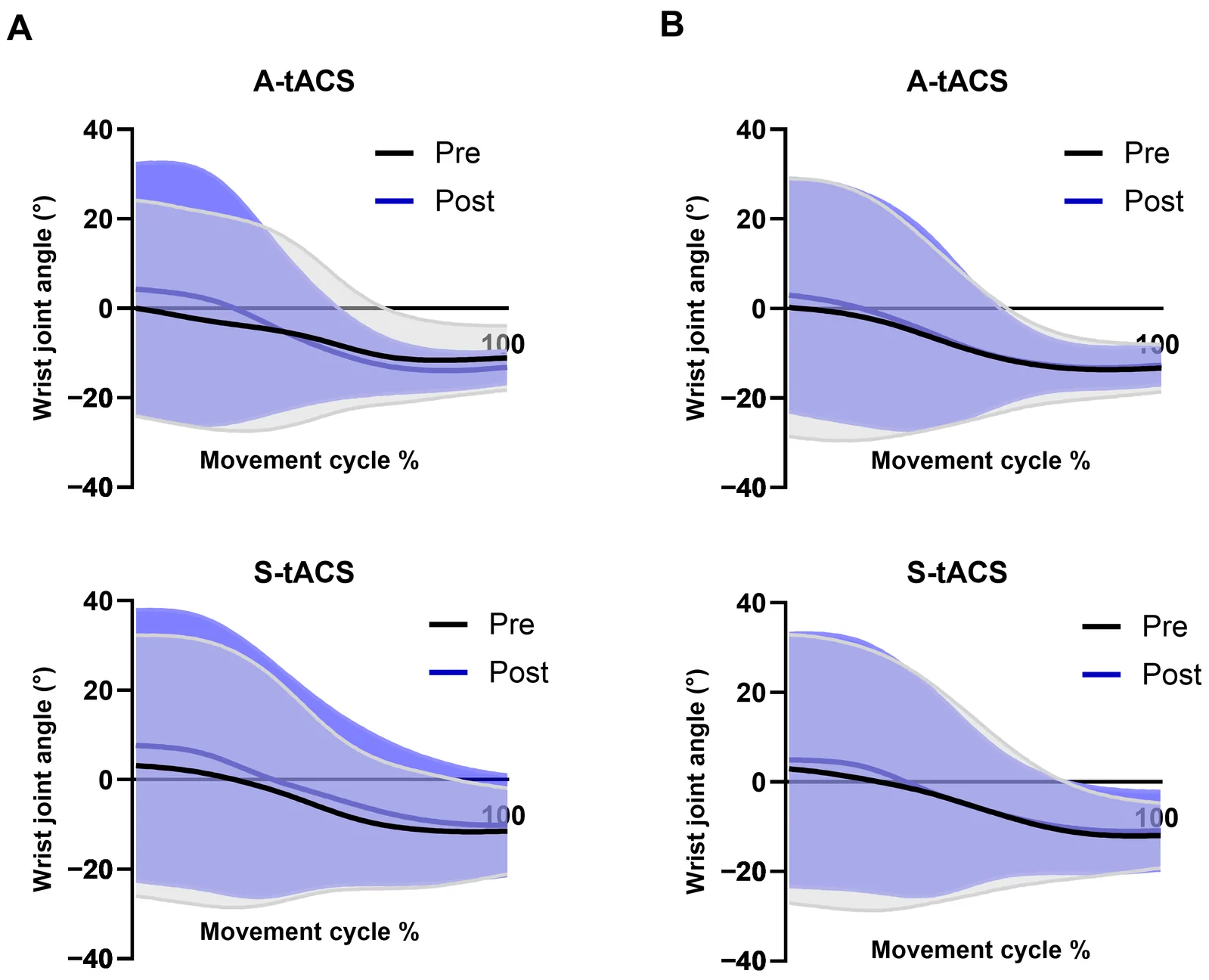
Wrist joint-angle trajectories before and after active and sham HD-tACS in the N1 and N2 tasks: (**A**) N1; (**B**) N2. Note. The study included 16 participants. Continuous curves represent group means, and the shaded regions represent standard deviations. No point-by-point statistical tests were performed on the continuous curves. Inferential analyses were based on the prespecified discrete kinematic or kinetic features extracted from the trajectories. Panels (**A**,**B**) represent the N1 and N2 tasks, respectively.

No significant Stimulation × Time interactions were observed for elbow or wrist range of motion (elbow: *p* = 0.229; wrist: *p* = 0.471; both BH-FDR-adjusted *q* = 0.781; Table 10). The corresponding Stimulation × Time × task interactions were also nonsignificant (both *p* ≥ 0.359), indicating no evidence that stimulation-related changes differed between N1 and N2. Adjusted marginal means and 95% CIs are presented in Table 11. A Period effect for elbow range of motion and a time×task interaction for wrist range of motion were observed, but these secondary effects do not represent stimulation-induced pre-to-post changes. The joint-angle trajectories before and after active and sham HD-tACS in the N1 and N2 tasks are presented in Figure 9 (elbow) and Figure 10 (wrist).

**Table 10:** Linear mixed-model tests for upper-limb kinematic and kinetic outcomes.

Outcome	Stimulation × Time, F (df)	*p*	BH-FDR *q*	Stimulation × Time × Task, F (df)	*p*	Other Significant Fixed Effect
Elbow ROM (°)	1.47 (1, 95.95)	0.229	0.781	0.85 (1, 95.77)	0.359	Period: F(1, 98.14) = 9.96, *p* = 0.002 †
Wrist ROM (°)	0.52 (1, 96.06)	0.471	0.781	0.08 (1, 96.04)	0.778	Time × Task: F(1, 96.05) = 4.55, *p* = 0.035 †
Elbow peak angular velocity (°/s)	0.30 (1, 96.08)	0.587	0.781	0.07 (1, 95.96)	0.796	Period: F(1, 97.68) = 7.17, *p* = 0.009 †; Stimulation × Task: F(1, 95.96) = 5.52, *p* = 0.021 †
Wrist peak angular velocity (°/s)	0.08 (1, 94.56)	0.774	0.781	2.21 (1, 94.41)	0.141	TaskPosition: F(2, 97.25) = 3.73, *p* = 0.028 †; Task: F(1, 94.39) = 18.24, *p* < 0.001 †
Peak elbow flexion moment (Nm/kg)	0.77 (1, 94.06)	0.384	0.781	0.20 (1, 94.03)	0.652	Stimulation × Task: F(1, 94.03) = 5.01, *p* = 0.028 †
Peak elbow extension moment (Nm/kg)	0.40 (1, 94.23)	0.529	0.781	0.14 (1, 94.11)	0.705	Stimulation × Task: F(1, 94.11) = 4.77, *p* = 0.032 †
Peak wrist flexion moment (Nm/kg)	0.08 (1, 94.99)	0.781	0.781	1.03 (1, 94.96)	0.313	—
Peak wrist extension moment (Nm/kg)	0.28 (1, 95.12)	0.595	0.781	0.57 (1, 95.08)	0.452	—

Note. *N* = 16. Unadjusted *p* values and Benjamini–Hochberg FDR-adjusted *q* values are reported for the eight prespecified Stimulation × Time tests. Bold text denotes statistically significant results. ROM = range of motion. * *q* < 0.05 (significant after BH-FDR correction). † Secondary or design-related effect; this should not be interpreted as evidence of a stimulation-induced pre-to-post change.

**Table 11:** Adjusted estimated marginal means (95% confidence intervals) for upper-limb kinematic and kinetic outcomes.

Outcome	Task	Sham Pre	Sham Post	Active Pre	Active Post
Elbow ROM (°)	N1	63.99 [55.50, 72.48]	62.41 [53.93, 70.89]	59.57 [51.32, 67.83]	67.36 [58.97, 75.76]
Elbow ROM (°)	N2	55.22 [46.74, 63.70]	58.21 [49.72, 66.70]	63.43 [55.18, 71.68]	67.70 [59.30, 76.09]
Wrist ROM (°)	N1	20.52 [9.77, 31.26]	24.46 [13.71, 35.20]	18.10 [7.41, 28.79]	24.70 [13.98, 35.42]
Wrist ROM (°)	N2	23.32 [12.58, 34.07]	22.32 [11.58, 33.07]	24.20 [13.51, 34.88]	24.36 [13.64, 35.08]
Elbow peak angular velocity (°/s)	N1	433.91 [378.08, 489.73]	436.36 [380.58, 492.14]	405.78 [351.17, 460.38]	428.97 [373.64, 484.30]
Elbow peak angular velocity (°/s)	N2	382.83 [327.05, 438.61]	400.49 [344.67, 456.31]	421.89 [367.31, 476.48]	446.93 [391.58, 502.28]
Wrist peak angular velocity (°/s)	N1	93.43 [54.74, 132.11]	131.20 [92.55, 169.86]	99.96 [62.30, 137.62]	102.85 [64.58, 141.12]
Wrist peak angular velocity (°/s)	N2	53.85 [14.20, 93.50]	49.20 [10.51, 87.88]	68.79 [31.14, 106.43]	87.67 [48.72, 126.63]
Peak elbow flexion moment (Nm/kg)	N1	−3.074 [−3.885, −2.262]	−3.056 [−3.868, −2.245]	−2.592 [−3.398, −1.787]	−2.887 [−3.696, −2.078]
Peak elbow flexion moment (Nm/kg)	N2	−2.850 [−3.667, −2.032]	−2.666 [−3.478, −1.855]	−3.000 [−3.806, −2.195]	−2.917 [−3.730, −2.104]
Peak elbow extension moment (Nm/kg)	N1	2.819 [2.220, 3.418]	3.073 [2.474, 3.671]	2.936 [2.351, 3.521]	2.902 [2.308, 3.495]
Peak elbow extension moment (Nm/kg)	N2	2.544 [1.933, 3.156]	2.530 [1.931, 3.129]	3.175 [2.590, 3.759]	3.088 [2.485, 3.690]
Peak wrist flexion moment (Nm/kg)	N1	−0.101 [−0.151, −0.051]	−0.131 [−0.180, −0.081]	−0.100 [−0.149, −0.051]	−0.113 [−0.162, −0.063]
Peak wrist flexion moment (Nm/kg)	N2	−0.111 [−0.161, −0.061]	−0.105 [−0.154, −0.055]	−0.112 [−0.162, −0.063]	−0.116 [−0.165, −0.066]
Peak wrist extension moment (Nm/kg)	N1	0.110 [0.057, 0.163]	0.134 [0.081, 0.186]	0.119 [0.067, 0.171]	0.124 [0.071, 0.176]
Peak wrist extension moment (Nm/kg)	N2	0.111 [0.058, 0.164]	0.114 [0.061, 0.166]	0.129 [0.077, 0.181]	0.134 [0.082, 0.187]

Note. *N* = 16. Values are adjusted estimated marginal means [95% CI]. Joint moments are normalized to body mass (N·m/kg). See Table 10 for statistical significance. Individual estimates should only be interpreted as significant if supported by their corresponding statistical comparisons. ROM = range of motion.

#### 3.2.2. Joint Angular Velocity

**Figure 11:**
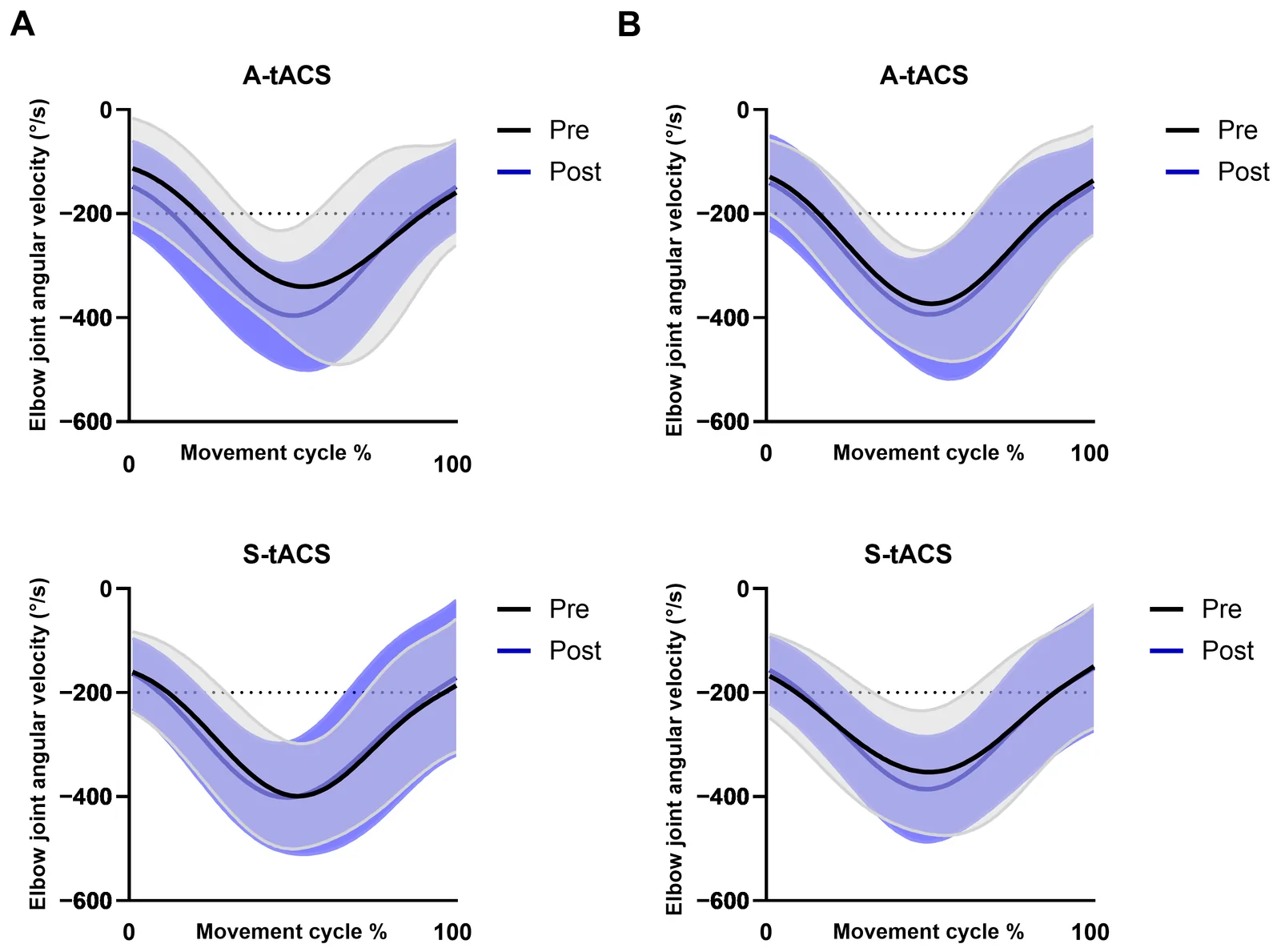
Elbow joint angular-velocity trajectories before and after active and sham HD-tACS in the N1 and N2 tasks: (**A**) N1; (**B**) N2. Note. The study included 16 participants. Continuous curves represent group means, and the shaded regions represent standard deviations. No point-by-point statistical tests were performed on the continuous curves. Inferential analyses were based on the prespecified discrete kinematic or kinetic features extracted from the trajectories. Panels (**A**,**B**) represent the N1 and N2 tasks, respectively.

**Figure 12:**
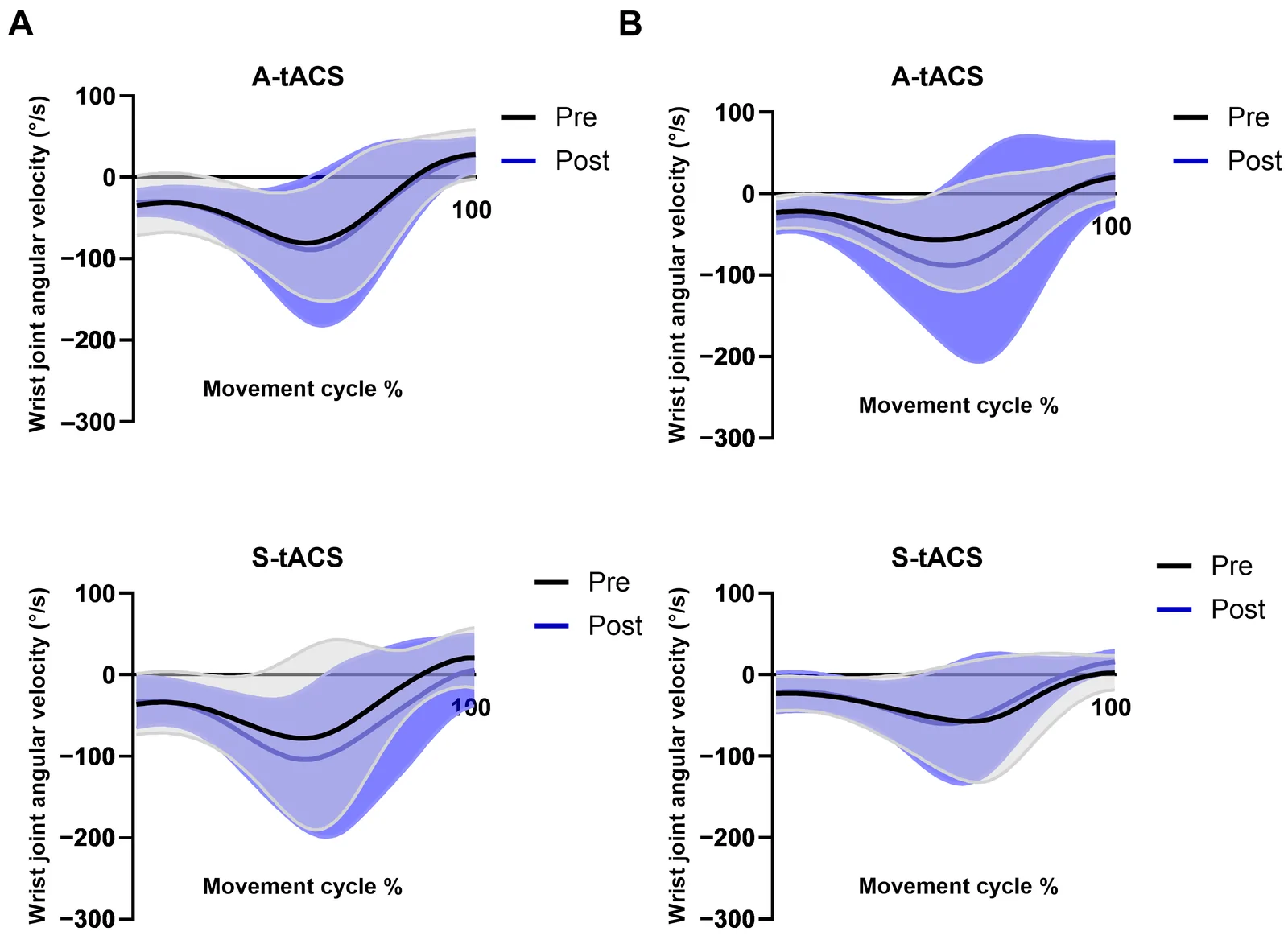
Wrist joint angular-velocity trajectories before and after active and sham HD-tACS in the N1 and N2 tasks: (**A**) N1; (**B**) N2. Note. The study included 16 participants. Continuous curves represent group means, and the shaded regions represent standard deviations. No point-by-point statistical tests were performed on the continuous curves. Inferential analyses were based on the prespecified discrete kinematic or kinetic features extracted from the trajectories. Panels (**A**,**B**) represent the N1 and N2 tasks, respectively.

No significant Stimulation × Time interactions were found for elbow or wrist peak angular velocity (elbow: *p* = 0.587; wrist: *p* = 0.774; both *q* = 0.781; Table 10). The corresponding three-way interactions were also nonsignificant (both *p* ≥ 0.141). Significant secondary effects involving Period, Task, TaskPosition, or stimulation-by-task were observed (Table 10), but none indicated a stimulation-induced pre-to-post change. Adjusted marginal means and 95% CIs are presented in Table 11. The joint angular-velocity trajectories before and after active and sham HD-tACS in the N1 and N2 tasks are presented in Figure 11 (elbow) and Figure 12 (wrist).

#### 3.2.3. Joint Moments

**Figure 13:**
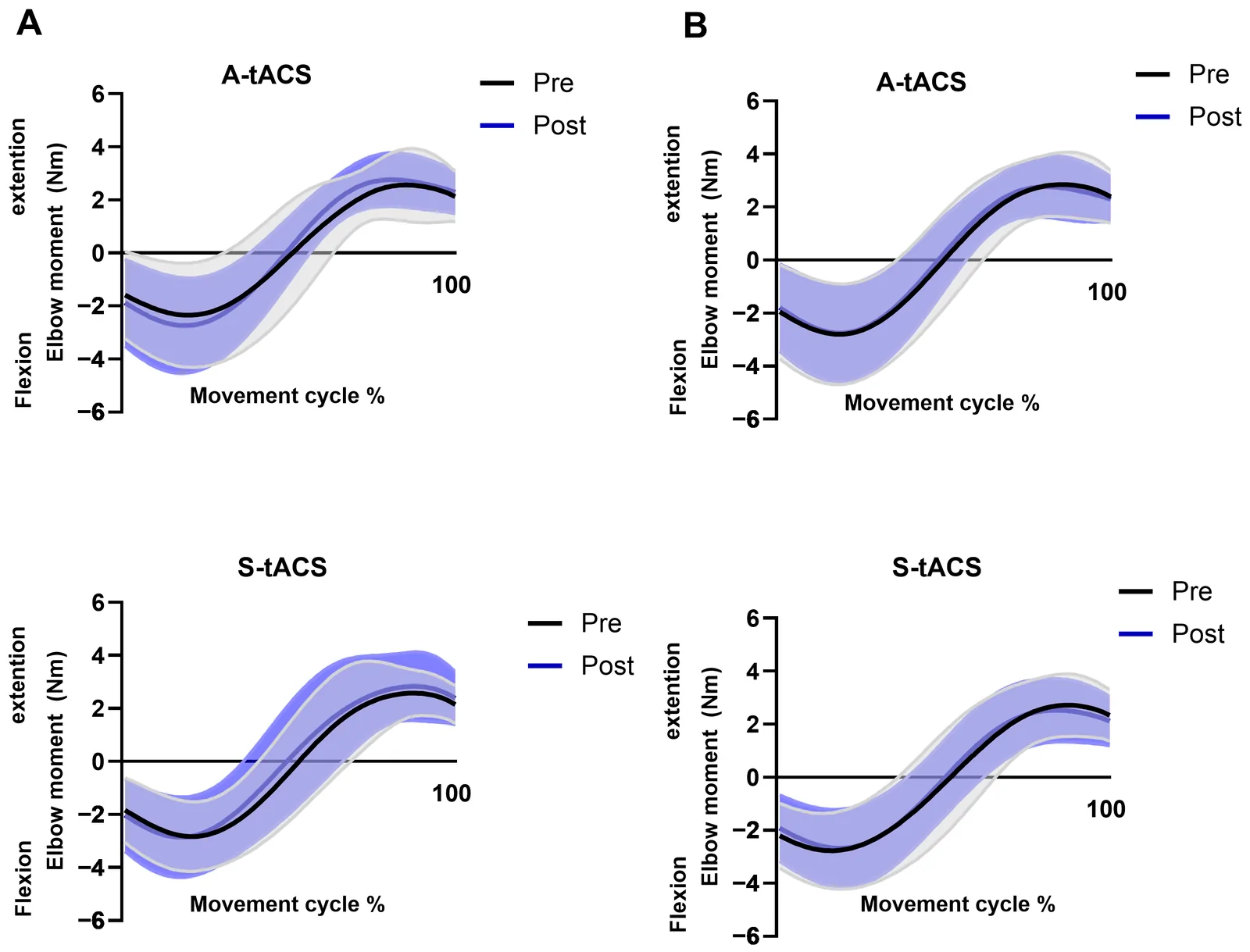
Elbow joint-moment trajectories before and after active and sham HD-tACS in the N1 and N2 tasks: (**A**) N1; (**B**) N2. Note. The study included 16 participants. Continuous curves represent group means, and the shaded regions represent standard deviations. No point-by-point statistical tests were performed on the continuous curves. Inferential analyses were based on the prespecified discrete kinematic or kinetic features extracted from the trajectories. Panels (**A**,**B**) represent the N1 and N2 tasks, respectively.

**Figure 14:**
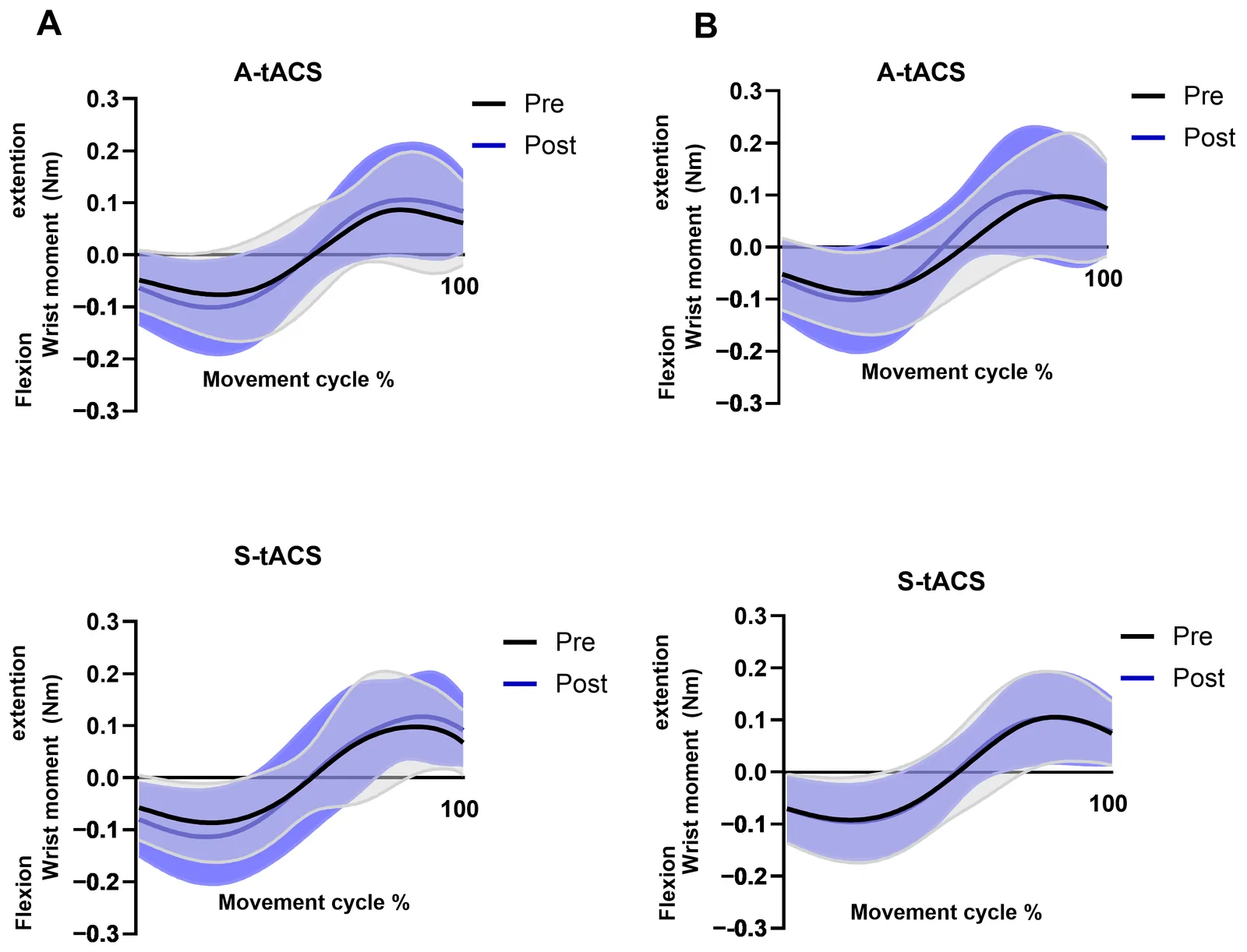
Wrist joint-moment trajectories before and after active and sham HD-tACS in the N1 and N2 tasks: (**A**) N1; (**B**) N2. Note. The study included 16 participants. Continuous curves represent group means, and the shaded regions represent standard deviations. No point-by-point statistical tests were performed on the continuous curves. Inferential analyses were based on the prespecified discrete kinematic or kinetic features extracted from the trajectories. Panels (**A**,**B**) represent the N1 and N2 tasks, respectively.

No significant Stimulation × Time interactions were found for peak elbow flexion, elbow extension, wrist flexion, or wrist extension moments (all *p* ≥ 0.384; all *q* = 0.781; Table 10). The corresponding three-way interactions were also nonsignificant (all *p* ≥ 0.313). Adjusted marginal means and 95% CIs are presented in Table 11. Although stimulation × task effects were observed for peak elbow flexion and extension moments, these effects were averaged across assessment times and therefore do not indicate stimulation-related changes. The joint-moment trajectories before and after active and sham HD-tACS in the N1 and N2 tasks are presented in Figure 13 (elbow) and Figure 14 (wrist).

## 4. Discussion

This study found a selective pattern of behavioral change after HD-tACS. Accuracy showed a significant Stimulation × Time interaction, with higher post-stimulation accuracy under active stimulation in both N1 and N2. Release-velocity DTC also showed a significant Stimulation × Time interaction across N1 and N2. In contrast, reaction time, raw release velocity, peak velocity, movement duration, and most upper-limb kinematic and kinetic outcomes showed no stimulation-related changes. The observed effects were therefore limited to selected behavioral outcomes.

### 4.1. Effects of tACS on Cognitive Task Performance

Accuracy and reaction time are commonly used indicators of cognitive task performance. Previous studies have reported that tACS may modulate both accuracy and reaction time [6,7]; however, findings remain inconsistent, possibly because of differences in stimulation frequency, stimulation site, task paradigm, and participant characteristics [26,27]. In the present study, active HD-tACS was associated with increased task accuracy in the N1 and N2 conditions, whereas no stimulation-related change in reaction time was detected. This dissociation suggests that accuracy and response speed may differ in their sensitivity to the present stimulation protocol; however, it should not be interpreted as a generalized improvement in cognitive–motor performance.

The present findings should also be considered in relation to mixed meta-analytic evidence. The finding is consistent with a literature that remains mixed. Meta-analyses in healthy adults have reported small or variable effects of tACS on cognition and working memory. The estimates depend on stimulation timing, frequency, cortical target, and cognitive domain, and some effects are reduced after correction for publication bias [6,7,28,29]. The present accuracy result is therefore best viewed as a protocol-specific finding that requires replication.

One possible explanation for this outcome pattern relates to the cortical target and stimulation frequency. The present study used a 4 × 1 high-definition montage targeting the dorsolateral prefrontal cortex (DLPFC), with stimulation delivered at 5 Hz in the theta band [24,25]. Debnath et al. [24] reported improved working-memory performance and increased EEG theta power following 5 Hz stimulation over the left DLPFC. The DLPFC is closely associated with working memory capacity, information maintenance, and information processing [30,31], and theta-band oscillations have been linked to information regulation during working memory tasks [32,33]. Nevertheless, the present study did not include EEG, fNIRS, TMS, or other neurophysiological measurements. It therefore remains unclear whether the observed change in accuracy was related to theta entrainment, modulation of DLPFC activity, or other working-memory-related processes. These mechanisms remain hypotheses for future studies that combine behavioral outcomes with direct physiological measurements.

### 4.2. Effects of tACS on Motor Task Performance

Release velocity, peak velocity, and movement duration were therefore selected as indicators of motor-task performance. However, no significant time × stimulation interactions were detected for these variables in the present study, indicating that the observed change in task accuracy was not accompanied by measurable changes in motor-task performance.

The characteristics of the present task should also be considered when interpreting this outcome pattern. Unlike a conventional N-back task based on a simple keypress response, the present paradigm required participants to retain and apply directional information and then execute a multi-joint throwing movement toward the selected target. Accuracy, reaction time, and movement kinematics therefore represented related but non-interchangeable components of performance. Upper-limb visuomotor studies by Ilardi et al. [17,18] similarly indicate that response accuracy, response speed, and movement characteristics capture distinct aspects of performance under cognitive demand, despite differences in populations and tasks. Accordingly, the fact that accuracy increased without corresponding changes in reaction time or kinematics suggests an outcome-specific behavioral change.

The stimulation target and frequency may also be relevant. The stimulation target in this study was the DLPFC rather than the primary motor cortex (M1), which is more directly involved in motor output [8,9]. Previous tACS studies aimed at modulating motor performance have often targeted M1 and used beta-band stimulation protocols [8,9]. Although brain regions are functionally connected and local stimulation may influence activity in distant areas [34], this study did not measure motor cortical excitability. Therefore, it cannot be determined whether DLPFC stimulation induced effective modulation of M1 activity.

This study initially assumed that improved cognitive performance under dual-task conditions might further contribute to improved motor performance. The present findings do not support this assumption. Instead, they suggest that the effects of tACS may depend on stimulation parameters, stimulation target, and task type. For a complex, multi-joint, goal-directed upper-limb movement such as throwing, whether cognitive enhancement alone can improve motor performance remains uncertain and requires further evidence [16].

### 4.3. Effects of tACS on Dual-Task Cost

Dual-task cost reflects the additional performance decrement that occurs when an individual performs a cognitive and a motor task simultaneously compared with performing a single task [10]. Based on the preliminary experimental results, release velocity, peak velocity, and movement duration were used to calculate dual-task cost in this study. Unlike previous dual-task studies that have mainly used walking or balance tasks, the present study combined an N-back working memory task with a throwing task, making cognitive judgment a prerequisite for motor execution. This design may better approximate real-world situations in which individuals must rapidly remember, judge, and execute a goal-directed movement in a complex environment.

The modulatory effect of tACS on dual-task cost was observed mainly in the more difficult N2 condition and was significant only for the dual-task cost of release velocity. Although the Holm-adjusted task-specific follow-up was significant for N2 but not for N1, the Stimulation × Time × Task interaction was not significant. The present data therefore do not demonstrate that the stimulation-related change was stronger under N2 or depended on N-back difficulty. Previous studies have also reported stronger effects of tACS during more demanding N-back tasks [24,25]. However, in the present dual-task paradigm, task difficulty was determined not only by the N-back level but also by the complexity of the throwing movement. Therefore, in cognitive–motor dual-task paradigms, task difficulty should not be defined solely by the N level; both cognitive load and motor task complexity should be considered.

This finding has implications for the design of dual-task training. For older adults or individuals with cognitive impairment, excessive task difficulty may make the task difficult to complete and thereby reduce the training effect. In contrast, for young adults with relatively preserved cognitive function, an appropriately challenging cognitive–motor dual-task paradigm may help avoid a ceiling effect in training. These implications, however, require further testing in populations with different ages and cognitive profiles.

### 4.4. Effects of tACS on Upper-Limb Movement Characteristics

No significant differences were observed in these variables before and after stimulation, indicating that the tACS protocol used in this study provided no evidence of stimulation-related changes in the measured upper-limb outcomes.

Two factors may help explain these findings. First, the stimulation protocol targeted the left DLPFC at 5 Hz and was primarily selected in relation to working-memory processing, rather than directly targeting M1 or another sensorimotor region. Previous studies examining motor outcomes have commonly applied beta-frequency tACS over M1. Wansbrough et al. [8,35] showed that the effects of beta-tACS over M1 on motor-cortical connectivity and sensorimotor power varied according to stimulation intensity and the physiological outcome examined. These findings highlight the target-, frequency-, intensity-, and outcome-specific nature of sensorimotor tACS and may help explain the absence of measurable changes in upper-limb kinematics following the present 5 Hz left-DLPFC protocol. Second, the angle, angular velocity, and moment profiles of the elbow and wrist joints showed substantial inter-individual variability, which may have reduced the detectability of stimulation effects at the group level [36,37,38]. Previous reviews have also suggested that the effects of non-invasive brain stimulation are influenced by individual neural state, anatomical characteristics, and stimulation parameters [39,40].

Future studies may therefore benefit from individualized brain modeling, neuroimaging, or electrophysiological measures to improve stimulation targeting and strengthen the interpretation of the findings. In addition, stimulation protocols involving multiple brain regions or multiple frequency bands may provide a possible direction for modulating cognitive–motor dual-task performance. Previous studies suggest that cross-frequency coupling and inter-regional phase synchronization may influence working memory performance [32,33,41]. Future research could further examine whether combined stimulation of the DLPFC and motor-related brain regions can modulate both cognitive processing and complex motor control.

### 4.5. Limitation and Future Directions

Several limitations should be noted. No single primary outcome was prespecified, and the power analysis assumed a large effect for the repeated-measures design. The analyses were therefore exploratory, and the study may have lacked power to detect small or moderate effects. Although false-discovery-rate correction was applied, the positive findings require replication in a study powered for a prespecified primary outcome.

The sample included only young male participants, which limits generalization to females and other age groups. The study also did not include EEG, fMRI, or another neurophysiological measure, so it cannot identify the neural mechanism underlying the behavioral changes. In addition, standardized adverse-effect and tolerability ratings and participant guesses about stimulation condition were not collected. Formal evaluation of tolerability and blinding success was therefore not possible.

Future studies should recruit larger, mixed-sex samples and combine the paradigm with direct physiological measurements. Trial-level analyses of speed–accuracy relationships and comparison with validated upper-limb visuomotor assessments may help establish the sensitivity of the task. Studies in older or clinical populations should first adapt the task difficulty to the target population.

## 5. Conclusions

This study used an integrated throwing-based cognitive–motor dual-task paradigm to examine behavioral and upper-limb motor outcomes after HD-tACS. Active stimulation was associated with higher accuracy and a favorable shift in release-velocity DTC. Most other outcomes showed no stimulation-related change. Overall, the findings support a limited, outcome-specific behavioral pattern rather than a generalized improvement in upper-limb motor performance.

## Figures and Tables

**Table 1:** Participant characteristics.

	Height (cm)	Weight (kg)	Age (year)
Mean ± SD	174.8 ± 5.4	78.5 ± 12.1	24 ± 2

Note. Values are presented as mean ± standard deviation; *n* = 16.

**Table 2:** Names and abbreviations of anatomical landmarks.

Landmark Name	Abbreviation
Superior border of the right iliac crest	RICST
Superior border of the left iliac crest	LICST
Right acromion	RSHO
Left acromion	LSHO
Right lateral elbow joint	REL
Right medial elbow joint	REM
Right lateral wrist joint	RWL
Right medial wrist joint	RWM
Third metacarpal of the right hand	HAND

**Table 3:** Definitions, calculation criteria, abbreviations, and units of outcome measures.

	Outcome Measure	Calculation Criterion	Abbreviation	Unit
Task-performance variables	Accuracy	The number of correctly directed throws divided by the total number of trials.	ACC	proportion
	Reaction time	The elapsed time from stimulus onset to movement onset for correctly directed trials, calculated as (movement-onset frame − stimulus-onset frame)/sampling frequency.	RT	s
	Movement duration	The elapsed time from movement onset to movement termination, calculated as (movement-termination frame − movement-onset frame)/sampling frequency.	MD	s
	Release velocity	The absolute value of the X-axis velocity component of the HAND marker at the identified object-release event. The absolute value was used because forward throwing corresponded to the negative X-axis direction.	RV	m/s
	Peak velocity	The maximum absolute value of the X-axis velocity component of the HAND marker over the entire movement cycle.	PV	m/s
	Dual-task cost	The percentage change in release velocity, peak velocity, or movement duration from the matched N0 reference to N1 or N2, calculated as [(dual-task value − matched N0 value)/matched N0 value] × 100.	DTC	%
upper-limb motor-control variables	Joint Range of Motion	The difference between the maximum and minimum joint angles in the specified plane over the entire movement cycle.	ROM	°
	Peak Joint Angular Velocity	The maximum joint angular velocity over the entire movement cycle.	V	°/s
	Peak Joint Flexion Moment	The maximum joint flexion moment over the entire movement cycle. Normalized by the body mass.	M-F	N·m/kg
	Peak Joint Extension Moment	The maximum joint extension moment over the entire movement cycle. Normalized by the body mass.	M-E	N·m/kg

**Table 9:** Adjusted estimated marginal means (95% confidence intervals) for dual-task-cost outcomes.

Outcome	Task	Sham Pre	Sham Post	Active Pre	Active Post
Release-velocity DTC (%)	N1	−8.99 [−27.79, 9.82]	−13.14 [−31.91, 5.63]	−12.15 [−30.66, 6.37]	12.39 [−6.15, 30.92]
Release-velocity DTC (%)	N2	−13.93 [−32.70, 4.84]	−7.77 [−26.58, 11.04]	−24.78 [−43.29, −6.26]	16.82 [−1.73, 35.38]
Peak-velocity DTC (%)	N1	1.89 [−12.11, 15.89]	12.15 [−1.82, 26.12]	−4.20 [−17.99, 9.58]	−3.66 [−17.46, 10.14]
Peak-velocity DTC (%)	N2	0.17 [−13.80, 14.15]	14.76 [0.76, 28.76]	−3.97 [−17.76, 9.81]	−1.33 [−15.14, 12.48]
Movement-duration DTC (%)	N1	6.07 [−3.72, 15.86]	10.02 [0.22, 19.82]	5.59 [−4.20, 15.38]	3.24 [−6.57, 13.04]
Movement-duration DTC (%)	N2	7.83 [−1.96, 17.61]	9.06 [−0.76, 18.88]	11.38 [1.59, 21.16]	5.33 [−4.49, 15.15]

Note. *N* = 16. Values are adjusted estimated marginal means [95% CI]. Dual-task cost was calculated as [(dual-task value − matched N0 value)/matched N0 value] × 100. A positive value denotes a higher metric during the dual-task condition compared to N0, with performance interpretations depending on the specific outcome. See Table 8 for statistical significance. Individual estimates should not be interpreted as significant without supporting statistical comparisons.

## Data Availability

The anonymized trial-level data and analysis materials supporting the findings of this study are available from the corresponding author upon reasonable request, subject to the conditions of the ethics approval and applicable data-protection requirements. No custom analysis scripts or code were used in this study.
